# Menopausal symptom burden in postmenopausal women with fibromyalgia

**DOI:** 10.1590/1806-9282.20261170

**Published:** 2026-11-09

**Authors:** Baki Derhem, Turgut Kültür, Şerife Çobankaya

**Affiliations:** 1 Kırıkkale University, Faculty of Medicine, Department of Family Medicine – Kırıkkale, Turkey.; 2 Kırıkkale University, Faculty of Medicine, Department of Physical Medicine and Rehabilitation – Kırıkkale, Turkey.

**Keywords:** Fibromyalgia, Menopause, Postmenopause, Chronic pain

## Abstract

**OBJECTIVE:**

The aim of this study was to compare menopausal symptom severity between postmenopausal women with and without fibromyalgia syndrome, and to estimate the independent association of fibromyalgia syndrome with menopausal symptom burden.

**METHODS:**

Comparative cross-sectional study of 80 postmenopausal women: 40 with fibromyalgia syndrome and 40 without. The primary outcome was the Menopause Rating Scale total score; pain, fatigue and sleep disturbance were rated on 0–10 visual analog scales. The association of fibromyalgia syndrome with Menopause Rating Scale total score was estimated by multivariable linear regression adjusted for age, body mass index, education and route of menopause.

**RESULTS:**

Menopause Rating Scale total score was 26.2±9.7 with fibromyalgia syndrome versus 12.9±6.1 in controls (mean difference 13.3 points, 95%CI 9.6–16.9; Hedges’ g=1.63; p<0.001). Severe symptoms (Menopause Rating Scale ≥17) occurred in 85.0% versus 27.5% (risk ratio 3.1; 95%CI 1.8–5.2). Fibromyalgia syndrome remained independently associated with Menopause Rating Scale total score after adjustment (β=13.13; 95%CI 9.32–16.94; p<0.001); further adjustment for pain, fatigue and sleep visual analog scales attenuated but did not abolish this association (β=10.11; 95%CI 6.07–14.16).

**CONCLUSION:**

Postmenopausal women with fibromyalgia syndrome carry a substantially greater menopausal symptom burden than women of comparable age and body mass index. This association was independent of measured demographic and reproductive characteristics, but its independence from the core features of fibromyalgia syndrome could not be established, given the overlap between the two symptom sets.

## INTRODUCTION

Fibromyalgia syndrome (FMS) is a chronic disorder characterized by widespread musculoskeletal pain accompanied by fatigue, non-restorative sleep, stiffness, and affective and cognitive disturbances. It affects an estimated 2–4% of the general population, with a marked female predominance, and its prevalence peaks between 40 and 60 years, coinciding closely with the menopausal transition^1,2^. Current evidence attributes the syndrome primarily to altered central nociceptive processing rather than to peripheral inflammation^1,3^.

The female predominance of FMS and the fluctuation of its symptoms with reproductive events have long implicated sex hormones in its pathophysiology. Estrogen modulates nociception both peripherally and centrally, declining estrogen levels are associated with a reduced pain threshold, and symptom exacerbation is documented during low-estrogen states such as the premenstrual phase and the postpartum period^1,2^.

Menopause is defined by the cessation of ovarian follicular activity and a sustained hypo-estrogenic state. Climacteric symptoms overlap substantially with the clinical picture of FMS, sharing musculoskeletal pain, fatigue, sleep disturbance, mood and cognitive complaints, which has led some authors to hypothesize that FMS may form part of the climacteric syndrome, or even be a characteristic manifestation of the climacterium^1,2,4^. Approximately one-quarter of women with FMS report more severe symptoms after menopause, and early and surgical menopause have been associated with the onset or aggravation of FMS^1,2^.

Beyond biological mechanisms, recent studies have highlighted substantial knowledge gaps and negative perceptions regarding menopause among climacteric women, which may influence symptom reporting and healthcare-seeking behavior^5^.

Existing work has approached this overlap only indirectly. Carranza-Lira and Villalobos Hernandez related FMS prevalence before and after menopause to climacteric complaints but did not quantify the symptom severity associated with FMS^6^. Cerón Lorente et al. compared postmenopausal women with and without FMS, but the outcome was functional status rather than climacteric symptomatology^7^. Gkouvi et al. related fibromyalgia impact to menopausal symptom severity within an FMS cohort, without a comparison group^8^. Climacteric symptom severity has therefore not been compared between postmenopausal women with and without FMS using a menopause-specific instrument with adjustment for the principal confounders. The aim of our study was to compare menopausal symptom severity between these two groups and to estimate the extent to which FMS is independently associated with it.

## METHODS

### Study design and setting

This comparative cross-sectional study was conducted in the Physical Medicine and Rehabilitation (PMR) and Family Medicine outpatient clinics of Kırıkkale University Faculty of Medicine Hospital between March and December 2024. It was approved by the Kırıkkale University Non-Interventional Research Ethics Committee (decision no. 2024.02.19) and conducted in accordance with the Declaration of Helsinki and written informed consent was obtained from all participants. Reporting followed the Strengthening the Reporting of Observational Studies in Epidemiology (STROBE) statement^9^.

### Participants

The FMS group comprised postmenopausal women diagnosed according to the 2016 revisions to the 2010/2011 American College of Rheumatology (ACR) criteria, recruited consecutively from the PMR clinics^10^. Controls were postmenopausal women without FMS attending the family medicine clinics. Participants were not individually matched; comparability for age and body mass index (BMI) was verified after data collection (Table 1).

**Table 1:** Demographic, reproductive, and menopause-related characteristics of the study groups.

	Fibromyalgia group (n=40)	Control group (n=40)	p
Age (years)	59.5 (53.0–62.0)	59.5 (55.0–61.3)	1.000
Body mass index (kg/m^2^)	28.3 (26.2–32.0)	28.1 (27.1–29.9)	0.973
Gravidity	3 (2–4)	3 (2–4)	0.759
Living children	2 (2–3)	3 (2–3)	0.328
Children in the same household	0 (0–1)	0 (0–0)	0.558
Married	32 (80.0)	36 (90.0)	0.348
High school education or above	9 (22.5)	8 (20.0)	1.000
Currently employed	6 (15.0)	4 (10.0)	0.735
Current smoker	6 (15.0)	6 (15.0)	1.000
Alcohol use	0 (0.0)	0 (0.0)	–
Natural menopause	37 (92.5)	36 (90.0)	1.000

Data are median (interquartile range) for continuous variables and n (%) for categorical variables. Continuous variables were compared using the Mann-Whitney U test; categorical variables using the chi-square test with Yates’ continuity correction, or Fisher's exact test where any expected cell count was below 5. Alcohol use was not tested because no participant in either group reported use.

Menopause was defined as the absence of menstruation for at least 12 consecutive months not attributable to another cause (natural) or as bilateral oophorectomy (surgical); both routes were eligible. Inclusion criteria were female sex, postmenopausal status and ability to provide informed consent. Exclusion criteria, applied to both groups, were any chronic systemic disease capable of independently causing widespread pain, systemic corticosteroid use, regular non-steroidal anti-inflammatory drug use within the preceding three months, current menopausal hormone therapy, and incomplete data.

### Outcome measures

The primary outcome was menopausal symptom severity, measured with the Menopause Rating Scale (MRS). The MRS comprises 11 self-administered items rated from 0 (no symptom) to 4 (very severe), grouped into somato-vegetative, psychological and urogenital subscales; the total score ranges from 0 to 44 and was classified as no or little (0–4), mild (5–8), moderate (9–16) or severe (≥17)^11^. The Turkish version was used; Can Gürkan reported a total-scale Cronbach's α of 0.84^12^. The prespecified outcome was the MRS total score, and only total scores were entered into the study database; item-level responses were therefore unavailable and subscale-level comparisons could not be made.

The severity of chronic widespread pain, fatigue and sleep disturbance was recorded in both groups on 0–10 visual analog scales (VAS); for sleep, higher scores denote greater disturbance. Age, BMI, educational level, employment, marital status, smoking, alcohol use, gravidity and route of menopause were recorded on a sociodemographic form.

### Sample size

The sample comprised all eligible women enrolled during the study period (40 per group). For a two-sided independent-samples t-test at α=0.05, this provides approximately 80% power to detect a Cohen's d of ≈ 0.63. To the best of our knowledge, since there has been no previous study comparing MRS scores between women with and without FMS, the number above is a sensitivity estimate for the sample obtained rather than a planning calculation.

### Statistical analysis

MRS total scores were normally distributed but variances were unequal; Welch's t-test was therefore used, and the mean difference is reported with its 95%CI and Hedges’ g. The Mann-Whitney U test served as a supporting analysis and for the VAS measures, which were not normally distributed; effect size r was computed as Z/than a planning calculation.th andd with the Hodges-Lehmann estimator. Categorical variables were compared with the chi-square test or Fisher's exact test; the proportion with severe symptoms was compared using the risk ratio, risk difference and odds ratio.

The independent association of FMS with the MRS total score was estimated using multivariable linear regression adjusted for age, BMI, educational attainment and route of menopause. Two prespecified exploratory analyses were performed: additional adjustment for pain, fatigue and sleep VAS scores, and a test of the group×VAS pain interaction. A two-sided p<0.05 was considered significant. Analyses were performed in IBM Statistical Package for the Social Sciences (SPSS) Statistics version 25 (IBM Corp., USA) and Python 3.11.

## RESULTS

A total of 80 postmenopausal women were included: 40 with FMS and 40 controls. The groups were comparable across all measured demographic, reproductive and menopause-related characteristics (Table 1).

The MRS total score was markedly higher in women with FMS (26.2±9.7) than in controls (12.9±6.1). Welch's t-test gave a mean difference of 13.3 points (95%CI 9.6 to 16.9; p<0.001), corresponding to Hedges’ g=1.63. The non-parametric analysis agreed: median 28.5 (18.8–34.3) versus 11.5 (10.0–17.0) (p<0.001; r=0.63), with a Hodges-Lehmann location shift of 13.0 points (Table 2).

**Table 2:** Symptom severity, menopausal symptom burden, and distribution of MRS severity categories.

	Fibromyalgia group (n=40)	Control group (n=40)	p	Effect estimate (95%CI)
VAS—pain (0–10)	8 (6–10)	5 (2–7)	<0.001	3.0 (2.0–4.0); r=0.51
VAS—fatigue (0–10)	8 (8–10)	7 (5–8)	0.002	1.5 (1.0–2.0); r=0.35
VAS—sleep disturbance (0–10)	8.5 (6–10)	6 (5–8)	<0.001	2.0 (1.0–3.0); r=0.37
MRS total score (0–44)				
Mean±SD	26.2±9.7	12.9±6.1	<0.001	13.3 (9.6–16.9); g=1.63
Median (IQR)	28.5 (18.8–34.3)	11.5 (10.0–17.0)	<0.001	13.0 (9.0–18.0); r=0.63
MRS severity category, n (%)			<0.001	
No or little (0–4)	0 (0.0)	3 (7.5)		–
Mild (5–8)	0 (0.0)	4 (10.0)		–
Moderate (9–16)	6 (15.0)	22 (55.0)		–
Severe (≥17)	34 (85.0)	11 (27.5)	<0.001	RR 3.1 (1.8–5.2); RD 57.5 (39.8–75.2); OR 14.9 (4.9–45.4)

The primary comparison of MRS total score used Welch's t-test (unequal variances); the effect estimate is the mean difference with Hedges’ g. Median-based rows are supporting analyses using the Mann-Whitney U test; the effect estimate is the Hodges-Lehmann location shift (median of all between-group pairwise differences) with its distribution-free 95%CI, and r was computed as Z/√N (0.10 small, 0.30 medium, 0.50 large). The overall distribution of severity categories was compared using the chi-square test; the proportion with severe symptoms using Fisher's exact test. CI: confidence interval; IQR: interquartile range; MRS: Menopause Rating Scale; OR: odds ratio; RD: risk difference (percentage points); RR: risk ratio; SD: standard deviation; VAS: visual analog scale.

Somatic symptom measures were also higher in the FMS group (Table 2): median VAS pain 8 (6–10) versus 5 (2–7), fatigue 8 (8–10) versus 7 (5–8), and sleep disturbance 8.5 (6–10) versus 6 (5–8) (all p≤0.002).

The distribution of MRS severity categories differed between groups (p<0.001; Table 2). No woman with FMS fell into the "no or little" or "mild" categories, whereas seven controls (17.5%) did. Severe symptoms (MRS≥17) were present in 34 women with FMS (85.0%) versus 11 controls (27.5%): risk ratio 3.1 (95%CI 1.8–5.2), risk difference 57.5 percentage points, odds ratio 14.9 (95%CI 4.9–45.4).

After adjustment for age, BMI, educational attainment and route of menopause, FMS remained independently associated with MRS total score (β=13.13; 95%CI 9.32–16.94; p<0.001; Table 3); no covariate was independently associated with the outcome. The model explained 43.4% of the variance (adjusted R^2^=0.396). Residuals were normally distributed (Shapiro-Wilk p=0.332); because the Breusch-Pagan test indicated heteroscedasticity, HC3 standard errors are reported. All variance inflation factors were below 1.2.

**Table 3:** Multivariable linear regression for Menopause Rating Scale total score and sensitivity of the fibromyalgia effect to progressive adjustment.

		β	95%CI	p	VIF
Panel A. Primary adjusted model					
	Fibromyalgia (vs. control)	13.13	9.32 to 16.94	<0.001	1.01
	Age (per year)	-0.12	-0.47 to 0.23	0.488	1.18
	Body mass index (per kg/m2)	-0.04	-0.37 to 0.29	0.814	1.03
	High school education or above	0.73	-5.24 to 6.70	0.809	1.18
	Natural (vs. surgical) menopause	5.02	-1.57 to 11.60	0.134	1.02
Panel B. β for fibromyalgia across models					R2
	Model A—unadjusted	13.25	9.64 to 16.86	<0.001	0.41
	Model B—primary adjusteda	13.13	9.32 to 16.94	<0.001	0.43
	Model C—Model B+pain, fatigue and sleep VASb	10.11	6.07 to 14.16	<0.001	0.55

Panel A: model R2=0.434; adjusted R2=0.396; overall F test p<0.001. Residuals were normally distributed (Shapiro-Wilk p=0.332). HC3 heteroscedasticity-consistent standard errors are reported throughout. aAdjusted for age, body mass index, education and route of menopause. bPain, fatigue and sleep are constitutive of the fibromyalgia case definition rather than downstream consequences of it; Model C therefore adjusts away part of the exposure and is presented as a deliberately conservative, over-adjusted sensitivity analysis. β: unstandardized regression coefficient; CI: confidence interval; VAS: visual analog scale; VIF: variance inflation factor.

The magnitude of the group difference was stable across progressive adjustment (Table 3): unadjusted 13.25 points (95%CI 9.64–16.86), adjusted 13.13 points. Adding pain, fatigue and sleep VAS scores attenuated the coefficient but it remained substantial (β=10.11; 95%CI 6.07–14.16), these variables accounting for 23.0% of the adjusted group difference.

The association between somatic symptom severity and MRS total score differed markedly by group. Among women with FMS, each one-point increase in VAS pain, fatigue and sleep disturbance was associated with increases of 2.83, 2.37, and 2.30 MRS points, respectively (all p<0.001), whereas the corresponding slopes among controls were −0.17, 0.18 and −0.03 (all p>0.6).

## DISCUSSION

Postmenopausal women with FMS reported a substantially greater menopausal symptom burden than controls of comparable age and BMI: 13.3 MRS points (Hedges’ g=1.63), essentially unchanged after adjustment (β=13.13). No woman with FMS fell into the "no or little" or "mild" category, and 85.0% were severely symptomatic versus 27.5% of controls.

These findings extend the evidence linking FMS and menopausal symptoms. Gkouvi et al. reported that fibromyalgia impact was associated with menopausal symptom severity among 169 women with FMS, but without a comparison group^8^. Musculoskeletal complaints are highly prevalent during the climacteric^2,13^, and chronic pain has been associated with clustering of menopausal symptoms in a British birth cohort^14^. Our controls were accordingly not symptom-free (27.5% severely symptomatic), which is consistent with recent findings that menopause-related knowledge gaps and fears may influence symptom perception and reporting^5^.

Ozcivit et al. proposed that FMS may itself be a characteristic symptom of the climacteric, given the overlap in age of onset, epidemiology, etiology and symptoms^4^. If that were so, comparing the groups would approach tautology. Our data are difficult to reconcile with the strong form of this reading: the groups were comparable in route of menopause, reproductive history and age; route of menopause was not independently associated with MRS score and the coupling between somatic symptoms and menopausal symptom burden was confined to the FMS group (p<0.001). These observations do not, however, exclude FMS being a manifestation of the climacteric in a susceptible subgroup: the confinement of this coupling is equally consistent with menopause acting as an amplifier within an already sensitized system.

A structural objection to any comparison of this kind is criterion contamination: three of the 11 MRS items—physical and mental exhaustion, sleep problems, and muscle and joint problems—enumerate symptoms that also constitute the ACR case definition of FMS, so part of the instrument may re-measure the exposure. An arithmetic ceiling bounds but does not eliminate this: even with a maximum score of 4 on every overlapping item in cases and 0 in controls, their joint contribution reaches 12 points, below the observed 13.3 and additivity is itself improbable under central sensitization.

The same consideration governs the exploratory model. Pain, fatigue and sleep are constitutive of the FMS case definition rather than consequences of it, so entering them alongside group membership is a deliberate overadjustment; even so, the coefficient attenuated only from 13.13 to 10.11 points. Because all variables were measured concurrently, no mediational interpretation is warranted. The attenuated but persistent coefficient after adjusting for pain, fatigue, and sleep (β=10.11) suggests that while these core FMS features account for approximately 23% of the observed group difference, the remaining excess burden likely reflects broader aspects of central sensitization—including affective, cognitive, and other somatic dimensions—rather than a menopause-specific burden independent of FMS. This interpretation aligns with the nociplastic pain framework, wherein central amplification enhances perception of all bodily sensations, including climacteric symptoms. Depression, anxiety and cognitive symptoms were not measured and could therefore not be included in the adjusted model.

FMS is the prototypical nociplastic pain condition, characterized by altered central nociceptive processing in the absence of tissue damage^3,15^, and central sensitization amplifies neural signaling to produce hypersensitivity extending beyond nociception^16^. If FMS entails a generalized lowering of the threshold at which bodily sensations are registered as distressing, climacteric complaints would be amplified in proportion to the degree of sensitization, precisely the coupling we observed. On this hypothesis-generating reading, the elevated MRS score reflects ordinary climacteric symptoms passing through an amplifying central filter.

Route of menopause was not independently associated with MRS total score, and women using menopausal hormone therapy were excluded by protocol. In the only randomized evidence in this population, Stening et al. allocated 29 postmenopausal women with FMS to transdermal 17β-estradiol or placebo for 8 weeks and found no effect on pain^17^. Estrogen deficiency therefore appears insufficient to explain the excess burden, reinforcing guidance that FMS and climacteric complaints be assessed as separate but coexisting entities^1^.

For primary care, severe menopausal symptoms are approximately three times as common in postmenopausal women with FMS as in their peers of comparable age and BMI. This burden is not an incidental finding to be dismissed but a potentially modifiable contributor to quality of life, and the MRS is a short instrument validated in Turkish that can be used to identify it.

Recent work has highlighted substantial knowledge gaps and fears among climacteric women regarding menopause and its management, with many women reporting inadequate information from healthcare providers^5^. In this context, the threefold higher prevalence of severe menopausal symptoms in postmenopausal women with FMS underscores an urgent need for targeted health education interventions. Digital platforms such as "Menopausando" have shown promise in improving menopause literacy^18^, and such approaches may be particularly valuable for women with FMS, who face compounded symptom burden and may benefit from integrated educational strategies addressing both conditions.

### Strengths and limitations

The principal strength is a comparison group of comparable age and BMI, permitting an estimate of the incremental burden associated with FMS. Several limitations warrant consideration. First, the single-center, cross-sectional design precludes causal inference; the observed association could reflect reverse ordering, shared neurobiological substrates or a bidirectional relationship. Second, cases were recruited from PMR clinics and controls from family medicine clinics; women with FMS are by definition care-seeking for pain, which may lower the threshold for endorsing any symptom and inflate the estimated difference. Third, item-level MRS data were not retained, precluding subscale-specific analyses—particularly of the urogenital subscale, which shares no items with the ACR criteria and would have offered the most direct test of criterion contamination. Fourth, depression and anxiety were not measured with validated instruments; both are prevalent in FMS, contribute to the MRS psychological subscale and represent the most plausible unmeasured confounders. Fifth, the ≥17 threshold for severe symptoms, while conventional, lacks formal validation in Turkish women. Sixth, the exclusion of hormone therapy users limits generalizability to the broader postmenopausal population. Finally, the sample was determined by the number of eligible women enrolled rather than by a planning calculation, and the study is underpowered for subgroup comparisons and for secondary analyses; these should be regarded as exploratory.

## CONCLUSION

The independence of this association from the core features of FMS could not be established with these data. The confinement of the somatic–climacteric coupling to the FMS group is consistent with central amplification, although the design cannot test this directly. Menopausal symptoms warrant explicit assessment in postmenopausal women with FMS, and longitudinal studies controlling for depression and anxiety are needed to establish directionality.

## Data Availability

The datasets generated and/or analyzed during the current study are available from the corresponding author upon reasonable request.

## References

[1] Vidal-Neira LF, Neyro JL, Maldonado G, Messina OD, Moreno-Alvarez M, Ríos C (2024). Climacteric and fibromyalgia: a review. Climacteric.

[2] Blümel JE, Palacios S, Legorreta D, Vallejo MS, Sarra S (2012). Is fibromyalgia part of the climacteric syndrome?. Maturitas.

[3] Fitzcharles MA, Cohen SP, Clauw DJ, Littlejohn G, Usui C, Häuser W (2021). Nociplastic pain: towards an understanding of prevalent pain conditions. Lancet.

[4] Ozcivit IB, Erel CT, Durmusoglu F (2023). Can fibromyalgia be considered a characteristic symptom of climacterium?. Postgrad Med J.

[5] Rodrigues MAH, Reis ZSN, Verona APA, Teixeira MC, Pappa GL, Soares JM (2025). Climacteric women's perspectives on menopause and hormone therapy: knowledge gaps, fears, and the role of healthcare advice. PLoS One.

[6] Carranza-Lira S, Hernandez IB (2014). Prevalence of fibromyalgia in premenopausal and postmenopausal women and its relation to climacteric symptoms. Prz Menopauzalny.

[7] Cerón Lorente L, García Ríos MC, Navarro Ledesma S, Tapia Haro RM, Casas Barragán A, Correa-Rodríguez M (2019). Functional status and body mass ındex in postmenopausal women with fibromyalgia: a case-control study. Int J Environ Res Public Health.

[8] Gkouvi A, Kontouli KM, Pardali EC, Patrikiou E, Lambrinoudaki I, Goulis DG (2026). Fibromyalgia and menopause: friends with benefits?. Maturitas.

[9] Elm E, Altman DG, Egger M, Pocock SJ, Gøtzsche PC, Vandenbroucke JP (2007). The Strengthening the Reporting of Observational Studies in Epidemiology (STROBE) statement: guidelines for reporting observational studies. Lancet.

[10] Wolfe F, Clauw DJ, Fitzcharles MA, Goldenberg DL, Häuser W, Katz RL (2016). 2016 Revisions to the 2010/2011 fibromyalgia diagnostic criteria. Semin Arthritis Rheum.

[11] Heinemann K, Ruebig A, Potthoff P, Schneider HP, Strelow F, Heinemann LA (2004). The Menopause Rating Scale (MRS) scale: a methodological review. Health Qual Life Outcomes.

[12] Gürkan Ö (2005). Menopoz semptomlarını değerlendirme ölçeği'nin Türkçe formunun güvenirlik ve geçerliliği. Hemşirelik Forumu Dergisi.

[13] Wright VJ, Schwartzman JD, Itinoche R, Wittstein J (2024). The musculoskeletal syndrome of menopause. Climacteric.

[14] Borra C, Hardy R (2025). The association between chronic pain and the clustering of menopausal symptoms: evidence from a British birth cohort study. Maturitas.

[15] Kosek E, Clauw D, Nijs J, Baron R, Gilron I, Harris RE (2021). Chronic nociplastic pain affecting the musculoskeletal system: clinical criteria and grading system. Pain.

[16] Nijs J, George SZ, Clauw DJ, Fernández-de-Las-Peñas C, Kosek E, Ickmans K (2021). Central sensitisation in chronic pain conditions: latest discoveries and their potential for precision medicine. Lancet Rheumatol.

[17] Stening KD, Eriksson O, Henriksson KG, Brynhildsen J, Lindh-Åstrand L, Berg G (2011). Hormonal replacement therapy does not affect self-estimated pain or experimental pain responses in post-menopausal women suffering from fibromyalgia: a double-blind, randomized, placebo-controlled trial. Rheumatology (Oxford).

[18] Ignácio MO, Martins MG, Portella CFS, Santiago AE, Zangirolami-Raimundo J, Baracat EC (2026). ‘Menopausando’: a digital health education platform for women in the transition to menopause and postmenopause. Climacteric.

